# Meta-analysis of the relationship between ocular and peripheral serum IL-17A and diabetic retinopathy

**DOI:** 10.3389/fendo.2024.1320632

**Published:** 2024-04-22

**Authors:** Xiaodong Li, Wei Qin, Xuewei Qin, Dandan Wu, Chenyuan Gao, Yinyue Luo, Mingchao Xu

**Affiliations:** ^1^ The First Affiliated Hospital of Guizhou University of Traditional Chinese Medicine, Ophthalmology, Guiyang, China; ^2^ Zhongshan Hospital of Traditional Chinese Medicine, Ophthalmology, Zhongshan, China; ^3^ Traditional Chinese Medicine Hospital of Meishan, Ophthalmology, Meishan, China

**Keywords:** IL-17A, diabetic retinopathy, inflammatory factor, system analysis, meta-analysis

## Abstract

**Purpose:**

A systematic evaluation and Meta-analysis were performed to determine the relationship between IL-17A levels in ocular aqueous and peripheral venous serum samples and diabetic retinopathy (DR).

**Methods:**

PubMed, Embase, Web of Science, and CNKI databases were searched from the time of library construction to 2023-09-20.The results were combined using a random-effects model, sensitivity analyses were performed to determine whether the arithmetic was stable and reliable, and subgroup analyses were used to look for possible sources of heterogeneity.

**Results:**

A total of 7 case-control studies were included. The level of IL-17A was higher in the Nonproliferative DR(NPDR) group than in the Non-DR(NDR) group [SMD=2.07,95%CI(0.45,3.68),P=0.01], and the level of IL-17A in the proliferating DR(PDR) group was higher than that of the NDR group [SMD=4.66,95%CI(1.23,8.08),P<0.00001]. IL-17A levels in peripheral serum and atrial fluid were significantly higher in NPDR and PDR patients than in non-DR patients in subgroup analyses, and detection of peripheral serum IL-17A concentrations could help to assess the risk of progression from NPDR to PDR. Sensitivity analyses suggested that the results of the random-effects arithmetic were stable and reliable. Subgroup analyses based on assay method and sample source showed that the choice of these factors would largely influence the relationship between IL-17A levels and DR.

**Conclusion:**

Elevated peripheral serum and ocular aqueous humor IL-17A levels in diabetic patients are associated with the risk of DR, IL-17A may serve as a potential predictor or therapeutic target for DR, and IL-17A may be an important predictor of inflammation for the progression of NPDR to PDR.

**Systematic review registration:**

https://www.crd.york.ac.uk/prospero/, identifier CRD42024532900.

## 1 Introduction

Diabetic retinopathy (DR) is one of the most common chronic complications of diabetes mellitus and is also a hemorrhagic fundopathy with the highest blindness rate among fundus diseases, mainly characterized by retinal microangiopathy and retinal neurodegenerative changes, and the current incidence of DR in the world, especially in the developed regions, is rising with the increase in the number of diabetic patients, which has brought about a heavy economic burden and pressure on public health (1). The incidence of DR is increasing with the increase in the number of diabetic patients worldwide, especially in developed countries. Although the pathogenesis of DR is closely related to long-term hyperglycemia, the specific pathogenesis of DR is very complex, and most current studies support that inflammation is one of the important mechanisms in the development of DR, especially chronic, low-grade inflammation plays an early and central role in the pathogenesis of DR (2). Retinal inflammation may induce increased retinal microvascular permeability, disruption of the blood-retinal barrier, and retinal ischemia and hypoxia leading to the formation of neovascularization and fibroproliferative membranes (3). Currently, clinical studies on the treatment of DR have focused on the intermediate and advanced stages of the disease with limited efficacy, such as intravitreal injections of corticosteroids or anti-vascular endothelial growth factor (VEGF) drugs, which inhibit the release of excessive inflammatory cytokines in the retina, and laser photocoagulation of the retina to ameliorate the hypoxia and neovascularization of the retina (4). Therefore, finding biomarkers or therapeutic targets that can predict initial DR is critical for timely prevention and treatment of DR and slowing down its progression.

Advances in technology have made protein and genomics research a current research hotspot. Many studies have been conducted on samples from patients with DR by high-throughput proteomics to identify biomarkers that predict the development of DR, including tear fluid, cornea, atrial fluid, lens, vitreous, retina, and serum. Several well-characterized DR biomarkers have been identified, such as VEGF, intercellular adhesion molecule-1 (ICAM-1), and interleukin-6 (IL-6) (5).

Recent studies have shown that IL-17A plays an important role in the pathogenesis and disease progression of DR, but the relationship between different sample sources and different levels of IL-17A and the development of DR is not conclusive, therefore, this study was conducted by systematically reviewing the levels of IL-17A in the intraocular and peripheral serum of patients with relevant DR, and utilizing Meta-analysis for the possible association between IL-17A levels and DR quantitative analysis, aiming to better guide clinical and research work.

## 2 Research information and methodology

### 2.1 Literature search

A comprehensive search of Pubmed, Web of Science, Embase Databases, and CNKI databases was performed to find case-control studies of the relationship between IL-17A levels and DR with the search terms “interleukin-17A” or “ IL-17A” combined with “DR” or “Diabetic Retinopathy”. The language was limited to English or Chinese, and the search was conducted from the time the databases were constructed to 2023-09, finally, the references that met the requirements were found manually.

### 2.2 Inclusion criteria

(1) The type of study is a case-control study. (2) Study data must include IL-17A concentrations. (3) Samples for study assays are derived from patients’ ocular tissues such as aqueous humor, vitreous humor, tears, crystals, or peripheral venous blood serum. (4) The language is limited to English or Chinese.

### 2.3 Exclusion criteria

(1) literature that did not specify the specific type of study; (2) duplicate publications; (3) literature on cohort studies, reviews, cross-sectional studies, case reports, and conference abstracts; (4) animal experiments, *in vitro* experiments, and cellular experiments; and (5) literature with incomplete information and data.

### 2.4 Literature screening and data extraction

Separately, two independent researchers eliminated duplicates by reading the titles, abstracts, and other information in the literature, excluding literature that was not a case-control study analysis, and reading the full text of potentially eligible literature to further eliminate ineligible literature (e.g., unavailability of data, etc.). Characteristics of included studies were extracted from the screened literature. Controversial or unidentifiable data were resolved through discussion or involvement of a third investigator.

### 2.5 Evaluation of the quality of the literature

The quality assessment of the included literature was scored according to the Newcastle-Ottawa (NOS) risk of a bias assessment tool for cohort studies recommended by the Cochrane Handbook for Systematic Evaluators, with a total of 9 points, and ≥7 being considered a high-quality study.

### 2.6 Statistical analysis

Meta-analysis was performed using Review Manager 5.4 software, and effect sizes were expressed as standardized mean difference (SMD) and its 95% CI. Statistical heterogeneity was analyzed using the χ2 test, with P ≤ 0.1 and I2>50% as significant heterogeneity between studies; heterogeneity was quantitatively described using I2, and fixed-effects models were used to combine effect sizes for I2 ≤ 50%, and vice versa, random-effects models were used to combine effect sizes. Subgroup analyses were performed according to the type of study. In addition, sensitivity analyses were performed to assess the impact of individual studies on the final effect. Funnel plots were used to assess potential publication bias. p<0.05 was considered a statistically significant difference.

## 3 Results

### 3.1 Results of the literature search

**Figure 1:**
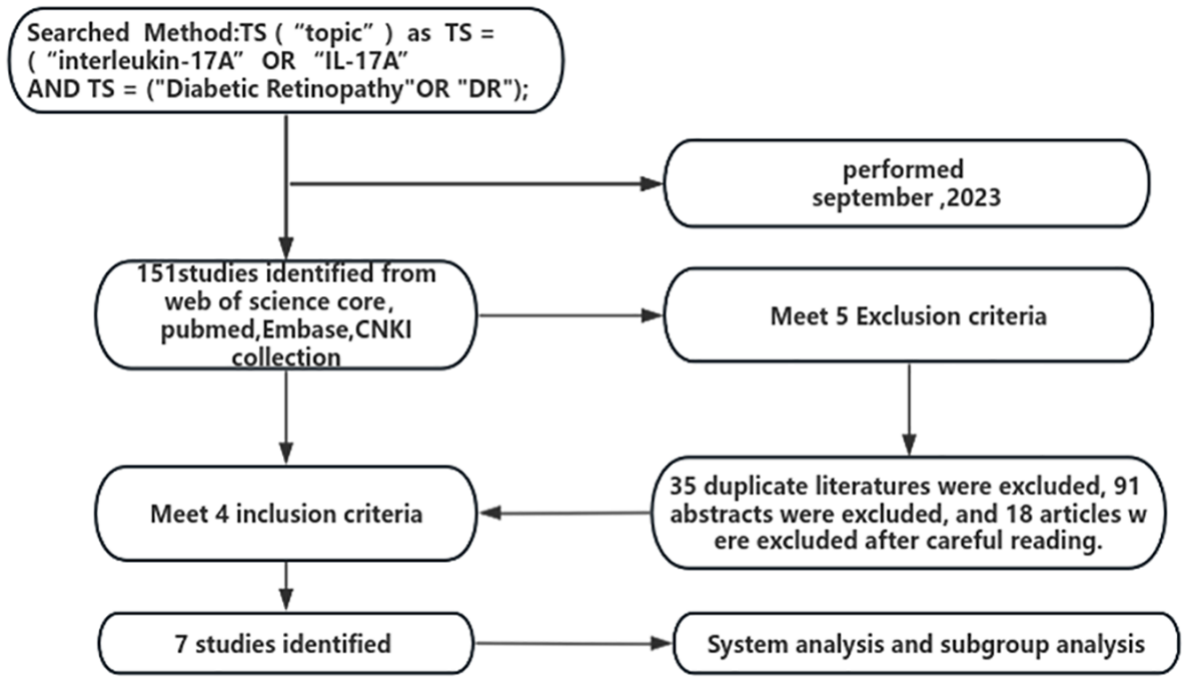
Flowchart of literature screening.

One hundred and forty-nine documents were retrieved, two documents were retrieved manually, 35 duplicates were excluded, 91 were excluded by reading the title abstracts, 18 were excluded by careful reading of the full text, and seven case-control studies were finally included. The literature screening process is shown in **Figure 1**. The basic characteristics of the included studies are shown in **Table 1**.

**Table 1:** Included in the study basic feature information table.

Studies	IL-17A levels			Sample size			Age		
	NPDR	PDR	NDR	NPDR	PDR	NDR	NPDR	PDR	NDR
Quevedo-Martínez JU (6)	47.4 ± 9.1	38 ± 7.3	50 ± 11.3	16	16	16	59 ± 8.5	56.9 ± 9.6	59.7 ± 11.2
Songfu Feng (7)	32.75 ± 0.68	35.78 ± 0.91	13.87 ± 0.42	11	9	20	/	/	58.4 ± 5.3
Nadeem Afzal (8)	375.95 ± 68.19	/	415.01 ± 83.4	152	/	30	50.88 ± 8.90	/	49.46 ± 9.94
Wang (9)	2.46 ± 0.22	/	2.37 ± 0.16	40	/	60	61.10 ± 11.37	/	58.51 ± 12.30
He (10)	9.24 ± 1.40	10.90 ± 1.76	6.92 ± 1.08	28	22	32	64.52 ± 6.76	65.35 ± 6.64	66.74 ± 6.83
Sun (11)	33.89 ± 6.32	/	21.20 ± 3.57	140	/	208	61.36 ± 13.31	/	58.93 ± 13.70
Zhang (12)	9.52 ± 1.30	10.89 ± 1.26	6.83 ± 1.02	34	30	36	62.70 ± 4.45	65.96 ± 6.36	69.86 ± 5.17

### 3.2 Literature quality evaluation results

The quality of the seven included studies was evaluated using the NOS scale applicable to case-control studies, and the results of the quality evaluation are shown in **Table 2**. The NOS scores indicate that the included studies were of high quality.

**Table 2:** Included in the study quality analysis table.

Author	Particular year	Type of Study	Sample Sources	Detection Methods	NOS
Quevedo-Martínez JU	2021	Original research	plasma	cytometric bead array Kit	8
Songfu Feng	2018	Original research	aqueous humor	ELISA	9
Nadeem Afzal	2014	Original research	plasma	ELISA	7
Chuan Wang	2015	Original research	plasma	ELISA	8
Donglin He	2021	Original research	aqueous humor	ELISA	7
Xiaofei Sun	2020	Original research	plasma	ELISA	7
Haijiang Zhang	2020	Original research	aqueous humor	ELISA	7

### 3.3 Meta-analysis results

#### 3.3.1 Meta-analysis of the relationship between IL-17A levels and DR

**Figure 2:**
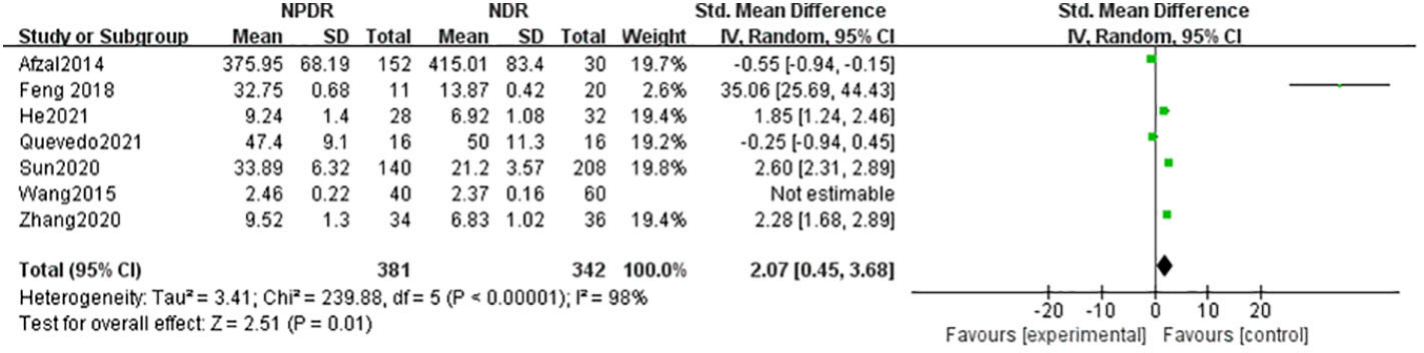
Forest plot of the relationship between IL-17A and NPDR.

**Figure 3:**
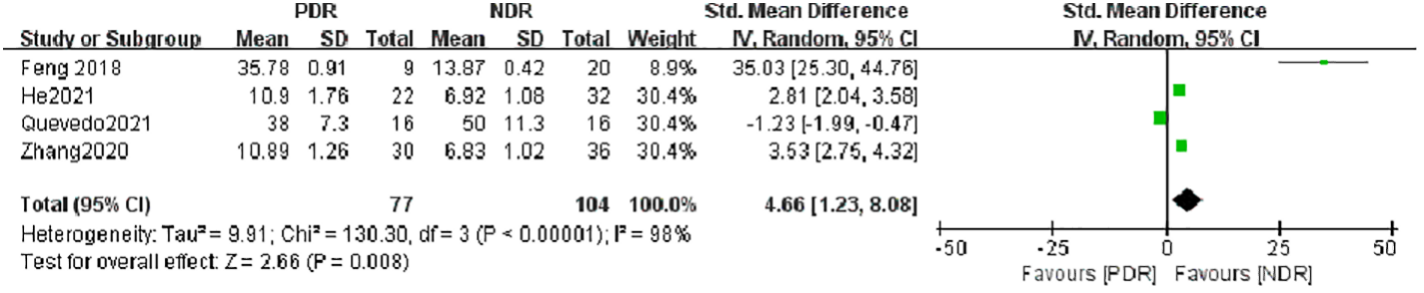
Forest plot of IL-17A about PDR.

The seven literatures of this study were tested for heterogeneity, I^2 ^= 98%,and P<0.01, suggesting that there is strong heterogeneity among the literature selected for this study, so random effects were selected for Meta-analysis, and the Meta-analysis results given by the random effects showed that the concentration of IL-17A in the NPDR group was higher than the concentration in the NDR group by 2.07,and it was statistically significant (P= 0.01), see **Figure 2**. The concentration of IL-17A in the PDR group was 4.66 higher than that in the NDR group and was statistically significant (P=0.008), see **Figure 3**.

#### 3.3.2 Sensitivity analysis

Sensitivity analysis was performed on the seven kinds of literature in the current study, analyzed by excluding individual studies one by one, comparing the SMD of IL-17A levels in the NDR group and the NPDR group at 1.07-2.07. The lower limit of the 95% CI was -0.13-0.69,the upper limit was 2.27-3.68,I^2^ at 97%-98%. Arbitrarily deleting the literature in the current study will not affect the results of the current study, implying that the results of the above random effects arithmetic are stable and reliable.

## 4 Subgroup analysis

### 4.1 Subgroup analysis based on sample source

Heterogeneity between serum (A) and atrial fluid (B) groups was high, I^2 ^= 98% (P < 0.01), indicating that the sample source would largely affect the results of a meta-analysis. The serum (A) group combined the results of 4 studies whose samples were derived from patients’ peripheral venous serum, and the IL-17A concentration in the NPDR group was slightly higher than that in the NDR group, with SMD=0.58, which was not statistically significant (Z=0.69, P=0.49), and the atrial fluid (B) group combined the results of 3 studies whose samples were derived from patients’ ocular atrial fluid, and the IL-17A in the NPDR group was significantly higher than that in the NDR group, with SMD=4.62, which was statistically significant (Z=0.69, P=0.49). SMD=4.62, which was statistically significant (Z=3.18, P=0.001). This suggests that the detection of ocular atrial fluid IL-17A is more helpful in detecting and predicting the progression of NDR to NPDR than the detection of serum IL-17A in patients with NDR. See **Figure 3**.

**Figure 4:**
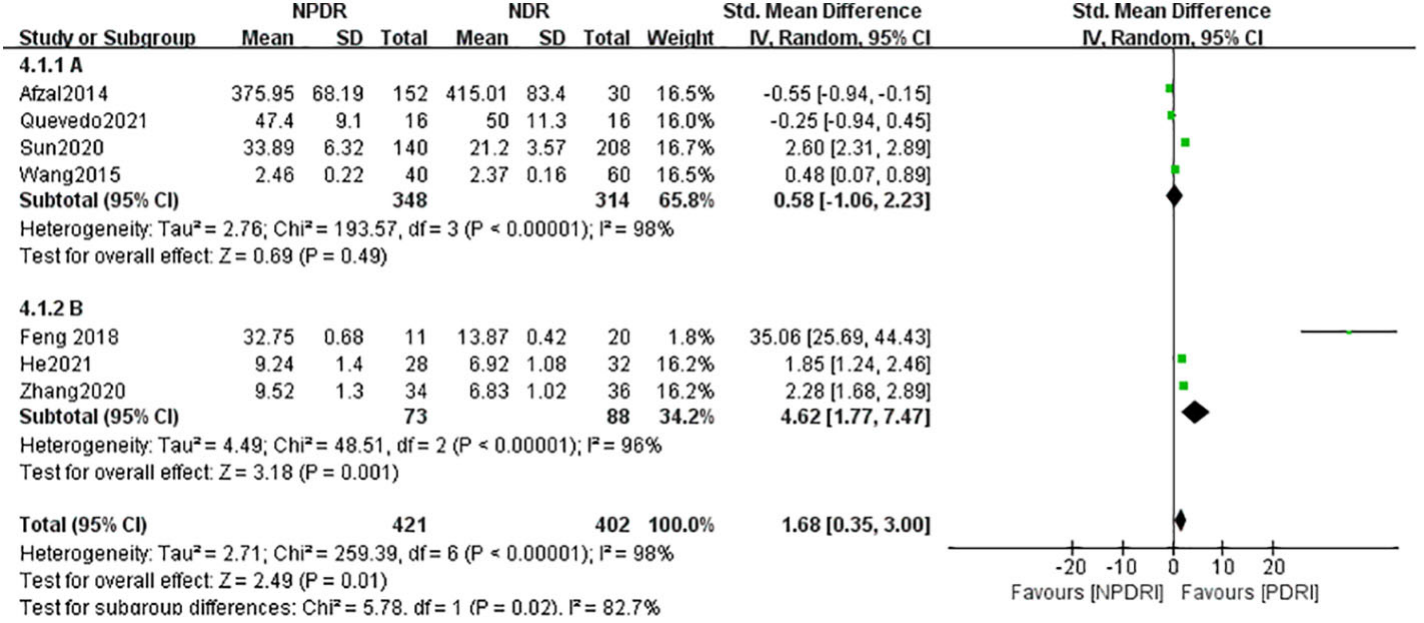
Subgroup analysis based on sample source (NDR-NPDR).

However, the serum (A) group combined the results of three studies whose samples were derived from patients’ peripheral venous serum, and the concentration of IL-17A in the PDR group was significantly higher than that in the NDR group, SMD=6.22, which was statistically significant (Z=3.61, P=0.0003), whereas the atrial fluid (B) group contained the results of only one study whose samples were derived from atrial fluid, and the concentration of IL-17A in the PDR group was lower than that in the NDR group, SMD=-1.23, which was statistically significant (Z=3.15, P=0.002). Since the results of the studies combined in the atrial water group were more homogeneous and highly heterogeneous, the results of this subgroup analysis can only indicate that detecting the concentration of serum IL-17A in patients with NDR can help to detect and predict the progression of NDR to PDR. See **Figure 4**.

**Figure 5:**
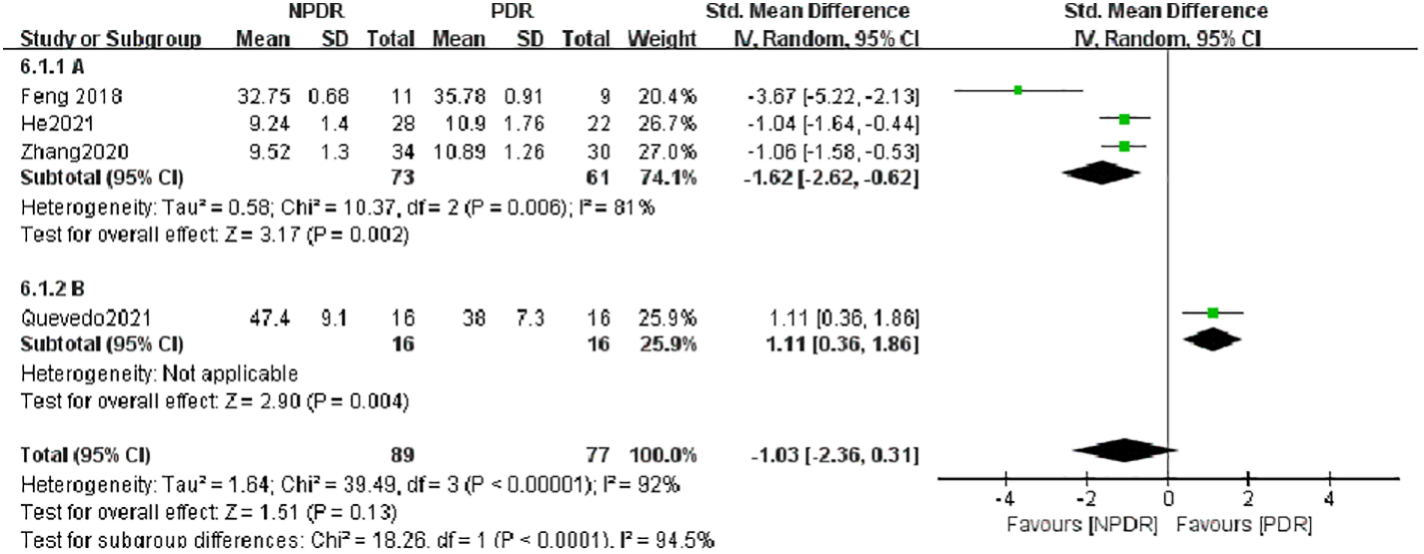
Subgroup analysis based on sample source (NDR-PDR).

Further analysis of the difference in IL-17A concentrations between the NPDR and PDR groups from different sample sources revealed that the concentration of IL-17A in the PDR group was significantly higher than that in the NPDR group in the serum (A) group, SMD=-1.62, (Z=3.17, P=0.002), whereas there was no statistically significant difference in the concentration of IL-17A between the PDR group and the NPDR group in the atrial fluid (B) group,. SMD=1.11 (Z=1.51, P=0.13) as shown in **Figure 5**. This suggests, in part, that testing patients’ serum IL-17A concentrations can be helpful in predictively assessing the risk of progression from NPDR to PDR.

### 4.2 Subgroup analysis based on assay method

**Figure 6:**
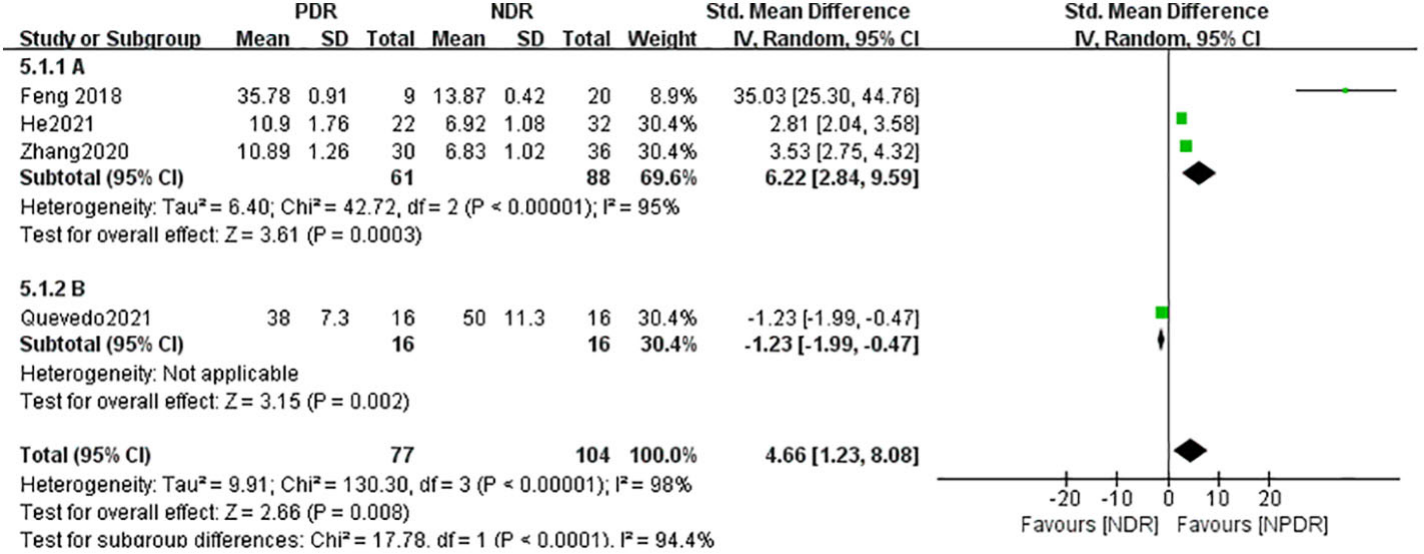
Subgroup analysis based on sample source (NPDR-PDR).

**Figure 7:**
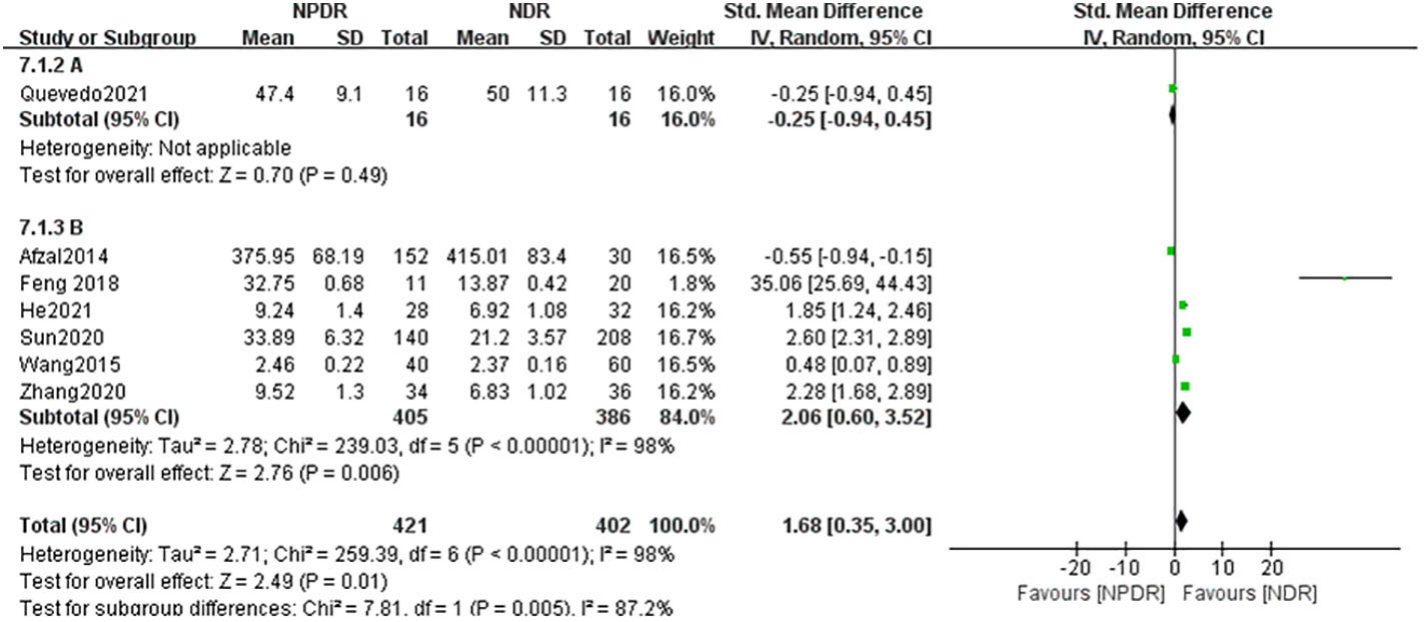
Subgroup analysis based on detection methods (NDR-NPDR).

**Figure 8:**
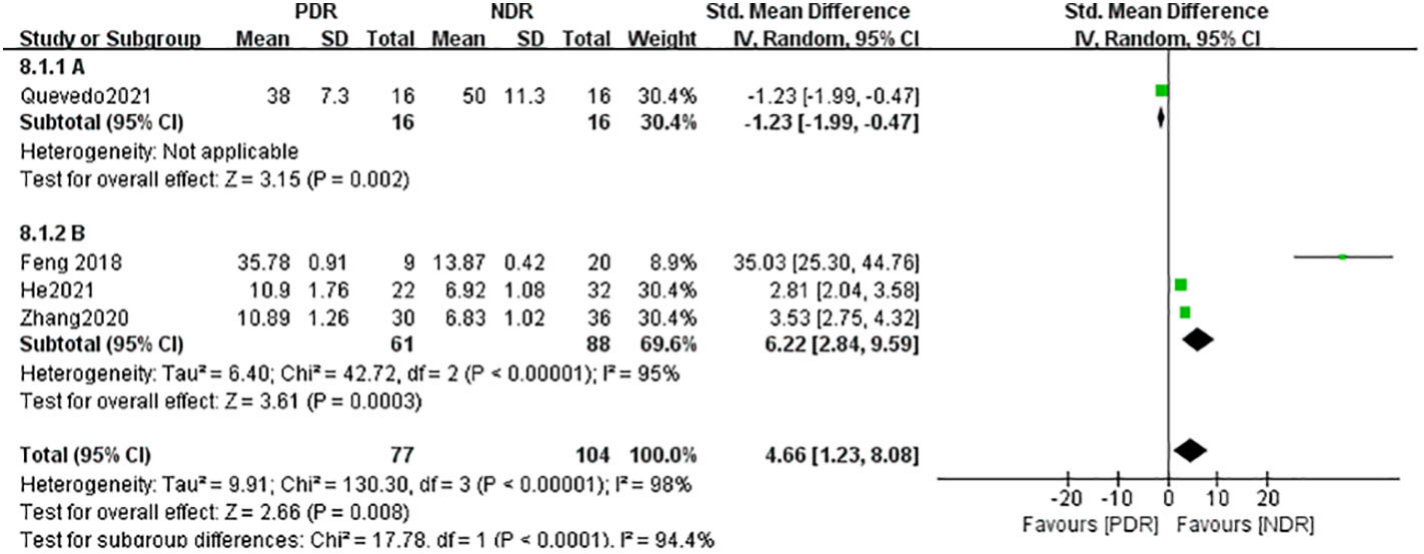
Subgroup analysis based on detection methods (NDR-PDR).

group A used the assay method cytometric bead array Kit, and the assay method used by Group B was ELISA, as shown in **Figure 6**, the heterogeneity between the two groups was high (I^2^ = 98%, P < 0.0001), indicating that the assay method would affect the results of meta-analysis to a certain extent, and the results of meta-analysis in group A (cytometric bead array Kit) included only one study, so no heterogeneity results were obtained; Group B (ELISA) included five studies and showed that IL-17A concentration in NPDR was significantly higher than that in the NDR group, SMD = 2.06, which was statistically significant (Z = 2.76, P = 0.006) but with higher heterogeneity (I^2^ = 98%, P < 0.00001). Again by analyzing the PDR and NDR groups with different assays, as shown in **Figure 7**, the heterogeneity between the two groups was high (I^2^ = 98%, P<0.00001), Group A (cytometric bead array Kit) still included only one study, so no heterogeneity results were obtained, and Group B (ELISA) included three studies, showing that PDR’s IL-17A concentration was significantly higher than that of the NDR group, SMD = 6.22 was statistically significant (Z = 2.66, P = 0.008), but heterogeneity was higher (I^2^ = 95%, P < 0.00001) as shown in **Figure 8**.

## 5 Publication of bias estimates

**Figure 9:**
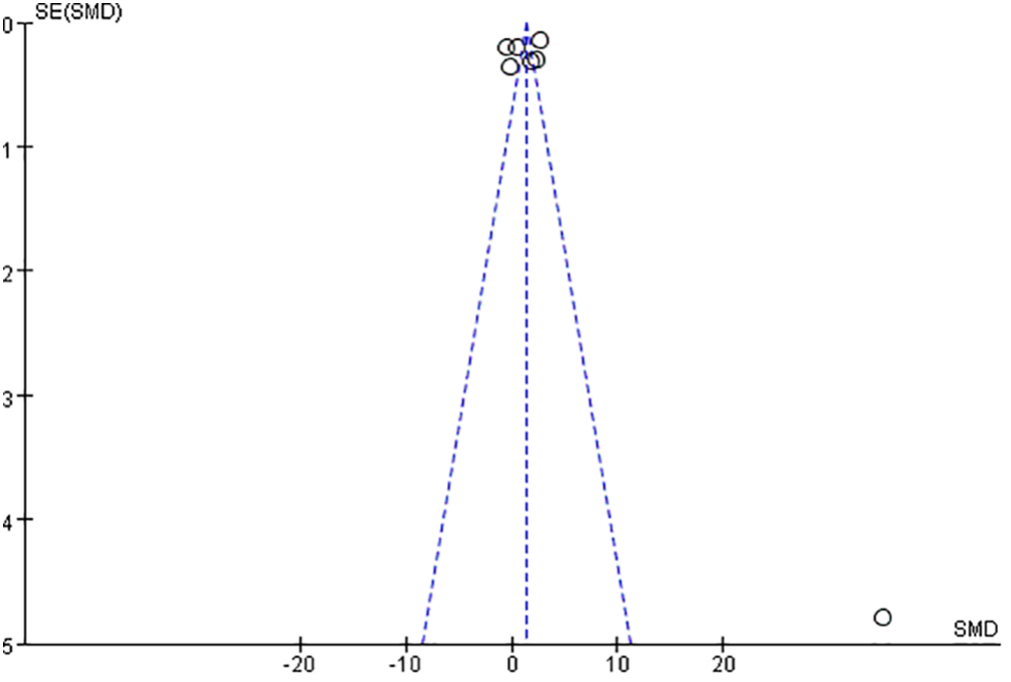
Funnel chart of included literature.

As shown in **Figure 9**, the funnel plot of the seven papers included in this study was symmetrical, and the bias test was also conducted to conclude that P>0.05, so it can be that there was no publication bias in the papers included in this study.

## 6 Discussion

DR is one of the most serious chronic neuro-microvascular complications of autoimmune diabetes, leading to vision loss and even loss of vision in diabetic patients, and has become a worldwide cause of blindness and incapacity in young adults with diabetes.IL-17A is a pro-inflammatory cytokine in Th17, which has been confirmed by many previous studies, and it is associated with a wide range of autoimmune diseases, and previous studies have showed that Th17 cells can cross the blood-retinal barrier through the circulatory system and participate in the inflammatory response in age-related macular degeneration (13) In this study, Th17 cells were shown to be involved in inflammatory responses in age-related macular degeneration (13), autoimmune uveitis (14, 15) and ischemic retinopathy (16). The production and increase of these inflammatory factors are closely associated with retinal Müller cells, astrocytes, and retinal pigment epithelial cells.IL-17A receptors are expressed on Muller glial cells, retinal endothelial cells, and photoreceptors (17). Several recent studies have shown that IL-17A induces an inflammatory response in retinal Müller cells and exacerbates apoptosis in retinal ganglion cells (18), which is involved in the onset and development of early DR (19). A previous animal study found that IL-17A exacerbates DR-like pathology by the promotion of Müller cell functional impairment via Act1//TRAF6/IKK/NF -κB signaling (20). It has also been shown that the IL-17A/IL-17R Act1/FADD signaling cascade leads to caspase-mediated apoptosis of retinal endothelial cells, confirming the pathological role of IL-17A in retinal capillary degeneration (21) which promotes microvascular case changes in DR. IL-17A overexpression inhibits cell proliferation and induces apoptosis, whereas IL-17A under expression accelerates cell proliferation and inhibits apoptosis. It was confirmed that miR-126 activated the PI3K-AKT pathway and decreased Bax and caspase-3 expression by targeting IL-17A, thus enhancing proliferation and inhibiting apoptosis in high glucose-induced retinal vascular endothelial cells (22). Recent studies have found that diabetes-mediated IL-17A enhances VEGF production in the retina, Muller glial cells, and retinal endothelial cells, and that IL-17A induces retinal endothelial cell proliferation and can enhance VEGF-dependent vascular neovascularization. Finally, a model of oxygen-induced retinopathy showed that IL-17A promotes retinal neovascularization (23). The current literature is more scattered in showing IL-17A as a possible cause of DR pathogenesis, but none of the more scientific studies have established the inevitability of their relationship. In this paper, we used Meta-analysis to systematically evaluate the correlation between ocular and peripheral blood serum IL-17A levels and DR in diabetic patients by grouping seven papers in terms of DR staging, and setting up subgroups in terms of measurement methods and sample sources, respectively.

The results of the current study showed that IL-17A levels were higher in NPDR patients than in NDR patients, with an effect size SMD=2.07,and statistically significant; IL-17A levels were significantly higher in PDR patients than in NDR patients, with an effect size SMD=4.66, and statistically significant. The sensitivity analysis suggested that the arithmetic results of random effects were stable and reliable. The demonstration that IL-17A is involved in the inflammatory immune response in DR is consistent with the findings of most current studies. Considering that the selection of factors such as measurement methods and sample sources may largely affect the results of Meta-analysis, subgroup analysis was performed. Analyzing from the sample source, the results of this study found that detecting the level of IL-17A in ocular atrial fluid of patients with NDR was more helpful in predicting the trend of progression to NPDR, but detecting the level of serum IL-17A in patients with NDR was helped predict the trend of progression to PDR and detecting the level of serum IL-17A in patients with NPDR was helped predict the assessment of the risk of progression to PDR. Analyzed in terms of measurement methods, the results of this study found that the ELISA was able to more accurately detect levels of ocular atrial fluid and peripheral serum IL-17A in patients with NDR, NPDR, and PDR. In a previous clinical study, flow cytometry and ELISA were used to detect IL-17A concentrations in the peripheral serum of 60 DR, 30 NDR and 30 healthy individuals, respectively. IL-17A levels in the vitreous humor of 31 PDR patients and 32 eyes with macular holes in the anterior retina were measured by ELISA after vitrectomy. Result I found that IL-17A concentrations in peripheral blood were significantly higher in patients with NDR compared with normal controls, and that IL-17A concentrations in peripheral blood of patients with DR decreased with the severity of DR and were negatively correlated with body mass index, duration of diabetes, and glycosylated hemoglobin. Results II found that vitreous fluid IL-17A levels were significantly higher in DR patients compared with controls (24). Different sample sources and different testing methods may affect the accuracy of the final results. Of course, with the progress of science and technology, there are more methods of high-throughput protein quantitative detection, which are currently limited to basic tests such as animal experiments and cellular experiments, due to the objective conditions of the limitations of the current clinical research is more convenient and quicker to use the ELISA method of detection, so the results of the present study will also be affected by this factor.

There are some limitations in this Meta-analysis: (1) there was a large heterogeneity among the included studies in the analysis of IL-17A levels and DR, so the confidence of the results may be reduced by combining the effect sizes only with a random-effects model; (2) the results of this study lacked samples from other parts of the eye, such as the vitreous fluid, due to the improved standard of uniformity of the data from the studies included in this study; (3) During the literature search process, individual relevant literature was inevitably omitted, and some literature could not be obtained with specific relevant data and was excluded; (4) The current included literature included only English and Chinese literature, and there was a certain degree of heterogeneity caused by the lack of language; (5) This study did not exclude the other influencing factors, such as age, living habits, cultural background, ethnicity, previous history of underlying diseases and other conditions that differed from the current study. history of underlying diseases, etc., which may cause differences in the results.

In summary, after systematic evaluation and Meta-analysis, it can be determined that IL-17A levels in ocular atrial fluid and peripheral serum of diabetic patients have a significant correlation with the development and severity of DR. Therefore, healthcare professionals should pay attention to strengthening the screening of IL-17A levels in patients with diabetes mellitus and early-stage DR, and searching for drugs that effectively inhibit IL-17A levels may be an important factor in the early screening of DR and intervention in the progression of early-stage DR. However, more multi-center and large-sample studies are needed to determine how high IL-17A levels lead to the onset of DR or the relationship with the rate of progression of DR.

## Data availability statement

The datasets presented in this study can be found in online repositories. The names of the repository/repositories and accession number(s) can be found in the article/supplementary material.

## Author contributions

XL: Writing – original draft, Writing – review & editing. XQ: Investigation, Writing – review & editing. DW: Data curation, Writing – review & editing. CG: Validation, Writing – review & editing. YL: Methodology, Writing – review & editing. WQ: Formal analysis, Validation, Visualization, Writing – review & editing. MX: Formal analysis, Validation, Supervision, Writing – review & editing.

## References

[1] Teo Z Tham Y Yu M Chee M Rim T Cheung N . Global prevalence of diabetic retinopathy and projection of burden through 2045: systematic review and meta-analysis. Ophthalmology. (2021) 128:1580–91. doi: 10.1016/j.ophtha.2021.04.027 33940045

[2] Forrester J Kuffova L Delibegovic M . The role of inflammation in diabetic retinopathy. Front Immunol. (2020) 11:583687. doi: 10.3389/fimmu.2020.583687 33240272 PMC7677305

[3] Rübsam A Parikh S Fort P . Role of inflammation in diabetic retinopathy. Int J Mol Sci. (2018) 19:1816. doi: 10.3390/ijms19040942 29565290 PMC5979417

[4] Wang W Lo A . Diabetic retinopathy: pathophysiology and treatments. Int J Mol Sci. (2018) 19. doi: 10.3390/ijms19061816 PMC6032159 29925789

[5] Youngblood H Robinson R Sharma A Sharma S . Proteomic biomarkers of retinal inflammation in diabetic retinopathy. Int J Mol Sci. (2019) 20:4755. doi: 10.3390/ijms20194755 31557880 PMC6801709

[6] Quevedo M Jonathan U Garfias Y Jimenez J Garcia O . Pro-inflammatory cytokine profile is present in the serum of Mexican patients with different stages of diabetic retinopathy secondary to type 2 diabetes. BMJ Open Ophthalmol. (2021) 6:e000717. doi: 10.1136/bmjophth-2021-000717 PMC8246380 34263060

[7] Feng S Yu H Yu Y Geng Y Dong L Chun Y . βLevels of inflammatory cytokines IL-1, IL-6, IL-8, IL-17A, and TNF- in aqueous humour of patients with diabetic retinopathy. J Diabetes Res. (2018) 2018:8546423. doi: 10.1155/2018/8546423 29850610 PMC5904804

[8] Afzal N Zaman S Asghar A Javed K Shahzad F Zafar A . Negative association of serum IL-6 and IL-17 with type-II diabetes retinopathy. Iranian J Immunol IJI. (2014) 11:40–8. 10.22034/iji.2014.16764 24632587

[9] Wang C Wang L Liu J Jun S Yu S Peng L . Irisin modulates the association of interleukin-17A with the presence of non proliferative diabetic retinopathy in patients with type 2 diabetes. Endocrine. (2016) 53:459–64. doi: 10.1007/s12020-016-0905-x 26940815

[10] He DL Jia MZ Tan YY Lin H . The value of atrial fluid IL-17, IL-23, and TNF-α levels in the diagnosis of diabetic retinopathy. J Med People's Liberation Army. (2021) 33:62–5.

[11] Sun XF Fan HJ Tian Y . Analysis of the relationship between serum 25-hydroxyvitamin D and IL-17 levels and diabetic retinopathy. J Mol Diagnosis Ther. (2020) 12:1349–52.

[12] Zhang HJ Liang L Tian R . Expression of IL-23 and IL-17 in atrial fluid of patients with diabetic retinopathy. Int J Ophthalmol. (2020) 20:1153–7.

[13] Chen J Wang W Li Q . Increased th1/th17 responses contribute to low-grade inflammation in age-related macular degeneration. Cell Physiol Biochem. (2017) 44:357–67. doi: 10.1159/000484907 29132135

[14] Zhong Z Su G Kijlstra A Yang P . Activation of the interleukin-23/interleukin-17 signalling pathway in autoinflammatory and autoimmune uveitis. Prog retinal eye Res. (2021) 80:100866. doi: 10.1016/j.preteyeres.2020.100866 32422390

[15] Li H Zhu L Wang R Xie L Ren J Ma S . Aging weakens Th17 cell pathogenicity and ameliorates experimental autoimmune uveitis in mice. Protein Cell. (2022) 13:422–45. doi: 10.1007/s13238-021-00882-3 PMC9095810 34748200

[16] Noueihed B Rivera JC Dabouz R Pénélope A Samy O . Mesenchymal stromal cells promote retinal vascular repair by modulating sema3E and IL-17A in a model of ischemic retinopathy. Front Cell Dev Biol. (2021) 9:630645. doi: 10.3389/fcell.2021.630645 33553187 PMC7859341

[17] Sigurdardottir S Zapadka TE Lindstrom SI Haitao L Brooklyn ET . Diabetes-mediated IL-17A enhances retinal inflammation, oxidative stress, and vascular permeability. Cell Immunol. (2019) 341:103921. doi: 10.1016/j.cellimm.2019.04.009 31076079 PMC6599623

[18] Qiu AW Huang DR Li B Fang Y Zhang WW Liu QH . IL-17A injury to retinal ganglion cells is mediated by retinal Müller cells in diabetic retinopathy. Cell Death Dis. (2021) 12:1057. doi: 10.1038/s41419-021-04350-y 34750361 PMC8575984

[19] Feng S Yu H Yu Y Geng Y Li D Yang C . Levels of inflammatory cytokines IL-1β, IL-6, IL-8, IL-17A, and TNF-α in aqueous humour of patients with diabetic retinopathy. J Diabetes Res. (2018) 2018:8546423. doi: 10.1155/2018/8546423 29850610 PMC5904804

[20] Qiu AW Bian Z Mao PA Liu QH . IL-17A exacerbates diabetic retinopathy by impairing Müller cell function via Act1 signaling. Exp Mol Med. (2016) 48:e280. doi: 10.1038/emm.2016.117 27980343 PMC5192073

[21] Lindstrom SI Sigurdardottir S Zapadka TE Tang J Liu H . Diabetes induces IL-17A-Act1-FADD-dependent retinal endothelial cell death and capillary degeneration. J Diabetes its complications. (2019) 33:668–74. doi: 10.1016/j.jdiacomp.2019.05.016 PMC6690768 31239234

[22] Chen X Yu X Li X Li L Li F Guo T . MiR-126 targets IL-17A to enhance proliferation and inhibit apoptosis in high-glucose-induced human retinal endothelial cells. Biochem Cell Biol = Biochimie biologie cellulaire. (2020) 98:277–83. doi: 10.1139/bcb-2019-0174 31608649

[23] Taylor BE Lee CA Zapadka TE Zhou AY Barber KG . IL-17A enhances retinal neovascularization. Int J Mol Sci. (2023) 24:1747. doi: 10.3390/ijms24021747 36675261 PMC9866094

[24] Chen H Ren X Liao N Wen F . Th17 cell frequency and IL-17A concentrations in peripheral blood mononuclear cells and vitreous fluid from patients with diabetic retinopathy. J Int Med Res. (2016) 44:1403–13. doi: 10.1177/0300060516672369 PMC5536736 27885039

